# The role of EGFR and ErbB family related proteins in the oligodendrocyte specification in germinal niches of the adult mammalian brain

**DOI:** 10.3389/fncel.2013.00258

**Published:** 2013-12-17

**Authors:** Alma Y. Galvez-Contreras, Alfredo Quiñones-Hinojosa, Oscar Gonzalez-Perez

**Affiliations:** ^1^Laboratorio de Neurociencias, Facultad de Psicologia, Universidad de ColimaColima, Mexico; ^2^Department of Neurological Surgery and Oncology, School of Medicine, Johns Hopkins UniversityBaltimore, MD, USA

**Keywords:** neural stem cell, oligodendrocyte, epidermal growth factor, platelet-derived growth factor, myelin, NG2 glia

## Abstract

In the adult brain, multipotent progenitor cells have been identified in three areas: the ventricular-subventricular zone (VZ-SVZ), adjacent to the striatal wall of the lateral ventricles, the subgranular zone (SGZ), located at the dentate gyrus of the hippocampus and the subcallosal zone (SCZ), located between the corpus callosum and the CA1 and CA2 regions of the hippocampus. The neural progenitor cells of these regions express the epidermal growth factor receptor (EGFR, ErbB-1 or HER1). EGF, the most important ligand for the EGFR, is a potent mitogenic agent that stimulates proliferation, survival, migration and differentiation into the oligodendrocyte lineage. Other ErbB receptors also activate several intracellular pathways for oligodendrocyte specification, migration and survival. However, the specific downstream pathways related to oligodendrogenesis and the hierarchic interaction among intracellular signaling cascades is not well-known. We summarize the current data regarding the role of EGFR and ErbB family signaling on neural stem cells and the downstream cascades involved in oligodendrogenesis in the neurogenic niches of the adult brain. Understanding the mechanisms that regulate proliferation, differentiation, migration of oligodendrocytes and myelination is of critical importance for the field of neurobiology and constitutes a crucial step in the design of stem-cell-based therapies for demyelinating diseases.

## Introduction

Neurogenesis, the production of new neurons, occurs throughout the life of adult mammals. This finding ended the dogma that prevailed since the times of Santiago Ramón y Cajal (at the beginning of the 20th century). He declared that neurons cannot be generated in adult brain and that astrocytes only provided support for brain parenchyma and neurons; therefore, the first discoveries of neuronal mitosis in adult brain were not well-accepted by the scientific community. To date, adult neurogenesis has been fully demonstrated in the adult mammalian brain (Doetsch et al., 1999b; Gritti et al., 1999; Sanai et al., 2004; Kriegstein and Alvarez-Buylla, 2009) and some evidence has been observed in the human brain (Eriksson et al., 1998; Sanai et al., 2004; Quinones-Hinojosa et al., 2006).

Adult neurogenesis has been extensively studied in two proliferative niches of the adult brain: the ventricular-subventricular zone (VZ-SVZ), adjacent to the striatal wall of the lateral ventricles (Kriegstein and Alvarez-Buylla, 2009) and the subgranular zone (SGZ), located at the dentate gyrus of the hippocampus (Seri et al., 2004). Recently, a third proliferative region referred to as the subcallosal zone (SCZ) is located between the corpus callosum and dorsal hippocampus. In these three regions, germinal cells have been isolated which produce multi-potential cellular conglomerates (neurospheres) *in vitro*. When seeded on adherent substrates, these multipotent neurospheres can generate neurons and glial cells (Seri et al., 2006; Laskowski et al., 2007). Yet, only gliogenesis has been found in the SCZ *in vivo*. Remarkably, the primary neural progenitor of these three regions has astrocytic characteristics, such as: clear cytoplasm, gap junctions, condensed chromatin and multiple cytoplasmic ramifications. In addition, these multipotent cells express molecular markers related to the astrocytic lineage as: glial fibrillary acidic protein (GFAP), nestin, vimentin, astrocytic glutamate transporter (GLAST) or calcium binding protein S-100β. Further, these cells express receptors with intra-cellular domains of tyrosine kinase receptors, such as: platelet-derived growth factor alpha (PDGFRα), fibroblast growth factor (FGFR) and epidermal growth factor (EGFR) (Garcia-Verdugo et al., 1998; Doetsch et al., 2002; Jackson et al., 2006; Seth and Koul, 2008; Kriegstein and Alvarez-Buylla, 2009).

The EGFR and its main ligand, EGF, are some of the most important mitogens that also have significant effects on the survival, migration and differentiation rate of embryonic (Aguirre et al., 2007; Chong et al., 2008; Hu et al., 2010; Sinor-Anderson and Lillien, 2011) and adult neural precursors (Gonzalez-Perez and Alvarez-Buylla, 2011). Recent studies in the adult brain indicate that EGFR stimulates the proliferation of neural precursor cells and induces their differentiation toward oligodendrocyte lineages (Gonzalez-Perez et al., 2009; Aguirre et al., 2010; Gonzalez-Perez and Quinones-Hinojosa, 2010). However, the precise mechanisms by which the EGFR and its ligands can induce such cellular differentiation are not entirely understood. The EGFR effects on differentiation seem to be driven by the heteromerization between the EGFR with other receptors (ErbB family receptor) and the subsequent signaling pathways activated by dimerization of them (Clark et al., 2012). This review summarizes the current information regarding the role of the ErbB signaling and the downstream signaling pathways involved in the oligodendrocyte specification in astrocytic neural stem cells. Additionally, we discuss neural disorders where the EGFR signaling in adult neural stem cells and oligodendrocyte precursors has been related to the pathophysiology of disease initiation and/or progression. Knowing the mechanisms that regulate proliferation, differentiation, migration and cell incorporation into the neural circuitry of stem-cell-derived oligodendrocyte progenitors is a crucial step in the design of therapies against demyelinating diseases.

## Neurogenic regions in the adult brain

The largest neurogenic regions in the adult brain of mammals are the VZ-SVZ and the SGZ. A third region (SCZ) seems to retain predominantly glyogenic neural progenitors. In these three regions, there are neural stem cells with multi-potential and self-regenerative characteristics *in vitro*; however, the *in vivo* properties of these progenitors are quite different. The VZ-SVZ generates diverse interneurons, that incorporate into the olfactory bulb (Doetsch et al., 1999a), and oligodendrocytes that migrate to adjacent white matter (Menn et al., 2006). The SGZ only generates neurons that incorporate in the granular cells layer of the dentate gyrus (Seri et al., 2001; Abrous et al., 2005), while the SCZ appears to generate only oligodendrocyte precursors that migrate and settle in the corpus callosum (Seri et al., 2006).

## Ventricular-subventricular zone (VZ-SVZ)

**Figure 1:**
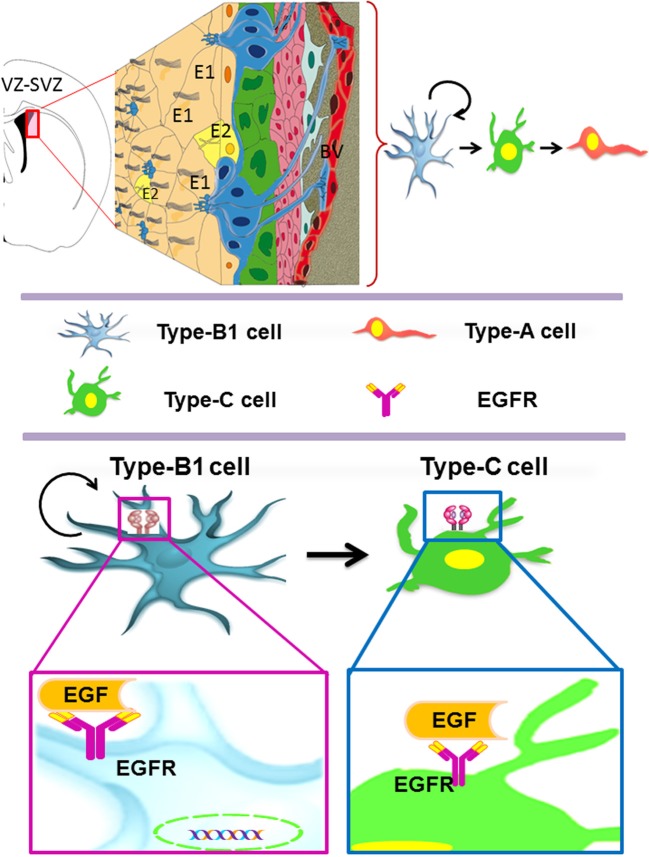
**The adult ventricular-subventricular zone (VZ-SVZ).** 3-D reconstruction of this niche of neural stem cells located within the lateral wall of the lateral ventricles. Multiciliated ependymal cells, also called E2 cells, form pinwheel-like structures (in peach color) around the apical processes of type B1 cells (in blue). Biciliated ependymal cells as referred to E1 cells (in yellow). Type-C cells (in green) and type-A cells (in red). Type-B1 progenitors are neural stem cells that generate secondary progenitors (type-C cells), which in turn give rise to migrating neuroblast (type-A cells). Additionally, type-B1 cells generate oligodendrocyte progenitors *in vivo*. Both type-B and type-C progenitors express the EGFR. Note that type-B neural stem cells are in close contact with the cerebrospinal fluid and the adjacent blood vessels (BV).

The adult VZ-SVZ is adjacent to the lateral walls of the lateral ventricles (Figure 1). This region has a complex cytoarchitecture delimitated by a layer of ependymal cells (E1- and E2-type cells) revisiting the ventricular cavity of the brain parenchyma (Mirzadeh et al., 2008). Adjacent to this layer of ependymal cells there are the type-B cells, which can be categorized in two subtypes: B1 cells and B2 cells. The B1 subtype are the *bona fide* neural stem cells (Doetsch et al., 1999b), while the B2 astrocytes appears to be supporting cells and constitute a limit between the neurogenic niche and the brain parenchyma (Mirzadeh et al., 2008). Type-B2 cells also form a net of glial tubes whereby the neuroblasts migrate toward the olfactory bulb (Lois and Alvarez-Buylla, 1994; Mirzadeh et al., 2008). Type-B1 cells have a cellular cycle of 17 h, with a 4.5-h length of S phase (Ponti et al., 2013). B1 cells also possess morphologic and ultrastructural characteristics of astrocytes (Doetsch et al., 1999b; Gritti et al., 1999) and express markers for several growth factors like PDGFRa, EGFR and FGFR-1 and -2 (Jackson et al., 2006; Frinchi et al., 2008; Danilov et al., 2009), as well as the class-IV intermediate filaments: vimentin, nestin and GFAP (Bonfanti and Peretto, 2007; Danilov et al., 2009).

The B1 cells give rise to transit-amplifying progenitor cells, also known as type-C cells (Figure 1). The type-C cells have a length of cell cycle between 18 and 25 h with a long S phase (12–17 h) and multiple cellular divisions (3 or 4 divisions) (Doetsch et al., 1999b; Gritti et al., 1999; Ponti et al., 2013). C cells express high levels of EGFR (Doetsch et al., 2002) and can be identified by the presence of the proneural gene Ascl1 (Ponti et al., 2013) and the transcription factor Dlx-2 (Danilov et al., 2009; Kriegstein and Alvarez-Buylla, 2009). Type C cells originate, in turn, type-A cells (migratory neuroblasts) that express the microtubular proteins doublecortin (DCX) and β-III tubulin (Tuj1), as well as the polysialylated neural cell adhesion molecule (PSA-NCAM) (Abrous et al., 2005; Bonfanti and Peretto, 2007). Type-A cells have a length of cell cycle of 18 h, with an S phase of 9 h (Ponti et al., 2013). These cells migrate tangentially through the rostral migratory stream (RMS) and reach the olfactory bulb (OB), where they disperse from the main cell stream, migrate radially and incorporate into the granular and periglomerular layers of the OB (Doetsch and Alvarez-Buylla, 1996; Alvarez-Buylla and Garcia-Verdugo, 2002; Hagg, 2005). Most of the SVZ-derived neuroblasts differentiate into GABAergic interneurons (Lois and Alvarez-Buylla, 1993, 1994), and a small percentage differentiate into dopaminergic (Basak and Taylor, 2009) or glutamatergic juxtaglomerular neurons that express the Neurog2, Trb1 and Trb2 transcription factors, as well as the vGluT1 and 2 glutamate-transporting proteins (Brill et al., 2009). The synaptic development and pruning of the VZ-SVZ-derived neurons are regulated by sensory activity (Saghatelyan et al., 2005) and experience-dependent plasticity long after maturation and integration (Livneh and Mizrahi, 2012). These newly generated neurons seem to function as neural mediators between sensory coding and brain plasticity (Adam and Mizrahi, 2010).

## The subgranular zone (SGZ)

**Figure 2:**
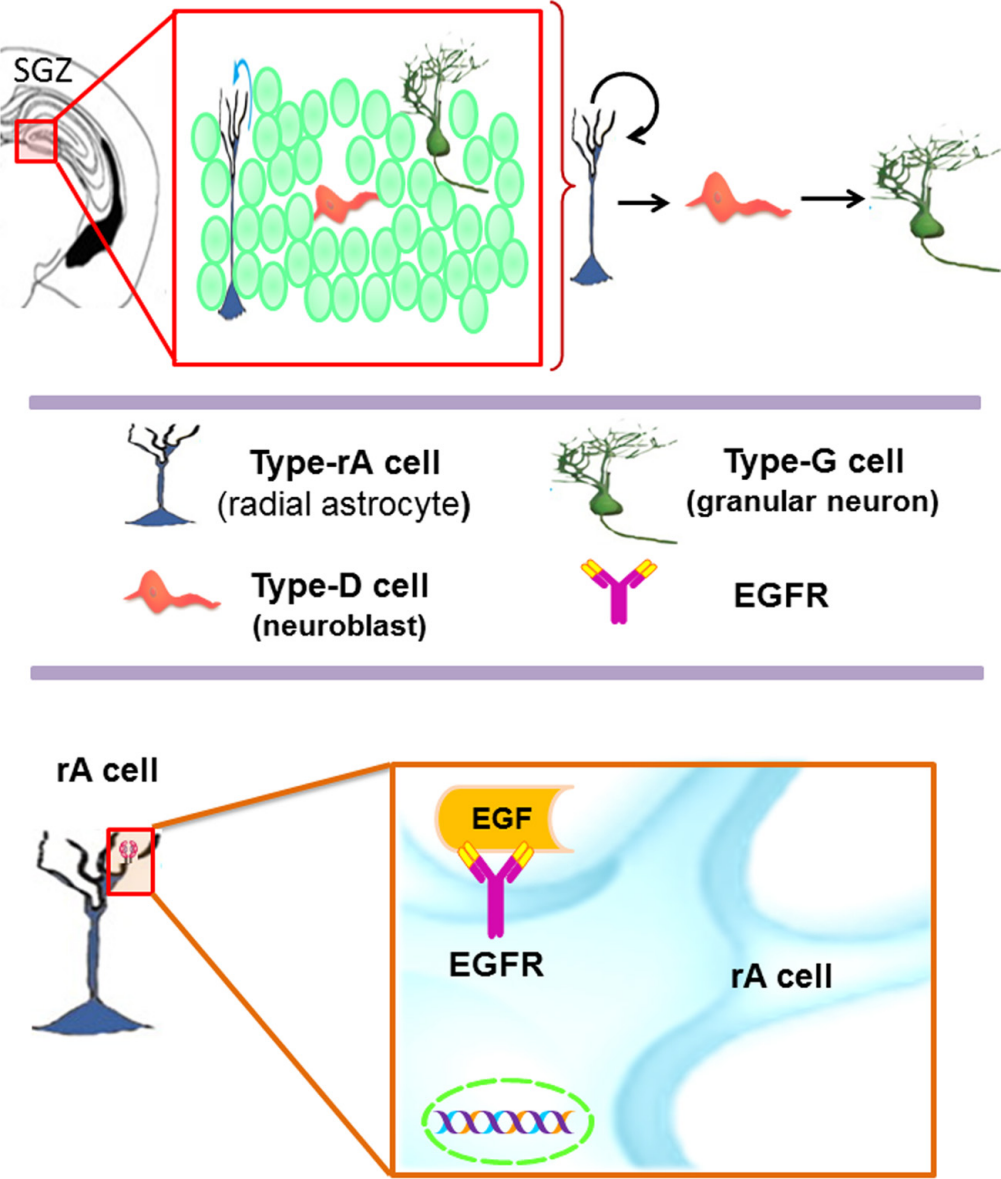
**The subgranular zone (SGZ) in the dentate gyrus of the adult hippocampus.** Type-B1 cells (in blue) also known as type-1 cells or type-rA cells (radial astrocytes) are the neuronal progenitor cells in this region. Type-rA cells divide and produce type-D cells as referred to type-2 cells. Hippocampal neuroblasts migrate locally and incorporate into the granular layer where they differentiate in mature granular neurons (type-G cells). Type-B1 cells express EGFR and behave as putative neural stem cells *in vitro*.

The SGZ is a neurogenic niche located in the dentate gyrus within the hippocampus (Figure 2) (Abrous et al., 2005). In the SGZ, there are two subtypes of astrocytes: the radial astrocytes and the horizontal astrocytes. The radial astrocytes, also known as type-1 cells, are the primary neuronal progenitors (Seri et al., 2001, 2004). Radial astrocytes express molecular markers as: GFAP, nestin, vimentin, S100β, musashi, 3-PGDH enzyme, and the transcription factor Sox-2 (Seri et al., 2001, 2004; Komitova and Eriksson, 2004; Balu and Lucki, 2009). SGZ astrocytes also express EGFR receptors, although in lower proportion as compared to the VZ-SVZ astrocytes (Jin et al., 2002). Radial astrocytes give rise to type-2 cells that are also referred as to type D cells (Seri et al., 2004). By their ultrastructural morphology, the type D cells can be subcategorized in type D1, type D2, and type D3, which express markers of immature neurons such as PSA-NCAM, DCX and Tuj1 (Seri et al., 2004; Lledo et al., 2006; Gonzalez-Perez et al., 2012). Type-D1 cells are small and ovoid. These cells give rise to D2 cell sub-type that possesses a short and thick process. According to the orientation of their cell processes, type D2 progenitors can be classified in three sub-types: D2v (vertically oriented), D2h (horizontally oriented) and D2i (pointing to the hilus) (Figure 2) (Seri et al., 2004). Type D3 cells have the morphology of immature granular neurons (Seri et al., 2004). Finally, the differentiation of type-D3 cells generates granular neurons (type-G cells) that can be identified by the expression of NeuN, calretinin, and calbindin (Ming and Song, 2005). Throughout this differentiation process, the new neurons of the SGZ migrate small distances (Hagg, 2005) and project their axons to CA3 neurons and synapse axonal projections from entorhinal cortex (Abrous et al., 2005; Balu and Lucki, 2009). Current evidence suggests that the hippocampal neurogenesis plays an important role in the spatial memory acquisition-retention process (Barnea and Nottebohm, 1996; Gould et al., 1999; Feng et al., 2001; Shors et al., 2001) and may regulate emotional processes such as stress, social behavior and depression (Warner-Schmidt and Duman, 2006; Gheusi et al., 2009).

## Subcallosal zone (SCZ)

**Figure 3:**
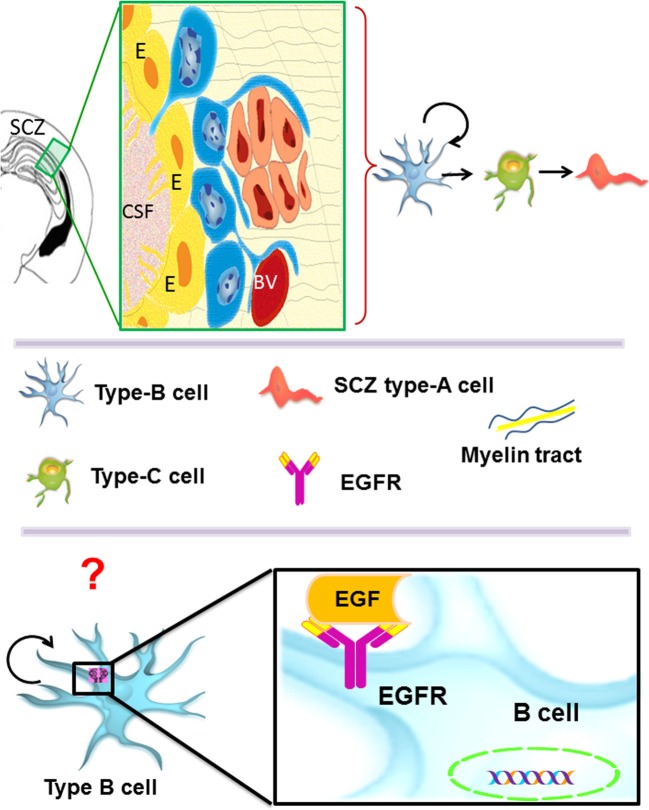
**The subcallosal zone (SCZ) is located between the hippocampus and the corpus callosum.** The SCZ is a caudal extension of the VZ-SVZ that is no longer associated to the ventricular system. Type-B cells (in blue) generate type-C cells that, in turn, give rise to oligodendrocyte precursors (also called SCZ type-A cells) that migrate into the neighboring corpus callosum. Type-B and type-C cells isolated from the SCZ and cultured as neurospheres behave as neural stem cells *in vitro*. However, the cell type that expresses *in vivo* the ErbB family receptors is unknown.

The SCZ is the most recently described germinal region and there is scarce information about it. Interestingly, its cellular composition is similar to the adult VZ-SVZ (Figure 3). During the embryonic development, the SCZ is formed by the collapsing of the ventricular zone walls. Nevertheless, in the adult brain, the SCZ is no longer associated with the ventricular system (Seri et al., 2006). The SCZ is comprised by several cavities filled with cerebrospinal fluid that are located between the corpus callosum and the hippocampus. The cytoarchitecture of the SCZ includes a layer of ependymal cells (type-E cells) that line each cavity. Type-E cells are frequently surrounded by myelinated and unmyelinated axons. Near the type-E cells, there is a subpopulation of astrocytes (type-B cells) and a small population of type-C cells. In this region, migratory cells (type-A cells) that express PSA-NCAM have been shown to differentiate into oligodendrocytes in the adjacent corpus callosum (Seri et al., 2006). Interestingly, the SCZ type-B cells originate multi-potential neurospheres when exposed to EGF or bFGF *in vitro* (Seri et al., 2006). A recent study indicates that the SCZ can generate neurons, but they cannot reach the mature stage (Kim et al., 2011). However, in mutant mice lacking Bax expression, the immature neurons derived from the SCZ are able to reach mature stages (Kim et al., 2011, 2012). In summary, the EGFR expression has been described in type B and type C cells of the VZ-SVZ and in the radial astrocytes of the SGZ. Although the SCZ cells can respond to the presence of EGF *in vitro* (Seri et al., 2006), the specific cell lineage that expresses EGFR is not well defined. This ability to respond to EGF indicates that the EGFR constitutes an important signaling pathway for the adult neural stem cells found in the VZ-SVZ, the SGZ and the SCZ.

## Receptor of the epidermal growth factor (EGFR)

The EGFR, also known as erbB1 or HER-1, is a glycoprotein with a molecular weight of 170 kd that belongs to the related proteins family of c-erbB. There are three other members of this family: erbB2/HER2, erbB3/HER3 and erbB4/HER4 (Table 1). The EGFR can generate homodimerization or heterodimerization with all ErbB family members (Yarden and Sliwkowski, 2001). After homo or heterodimerization, the tyrosine kinase intracellular domains of ErbB monomers self-phosphorylate and recruit several proteins that, in turn, activate downstream signaling pathways with various cell functions (Schneider et al., 2008). Thus, the EGFR regulates proliferation, migration, tissue invasiveness, apoptosis inhibition, cell differentiation, circadian rhythm, puberty initiation and development of cognitive functions (Herbst and Bunn, 2003; Liu and Neufeld, 2004; Brandes et al., 2008).

**Table 1:** **ErbB family members**.

**ErbB family member**	**Molecular weight**	**Description**	**Biological assembly**
ErbB1, HER1 or EGFR	170 kDa	EGFR is the main member of the ERBB receptor tyrosine kinase family. It promotes protein tyrosine kinase activity due to an induced dimerization of the receptor, an essential part of the signal transduction pathway.	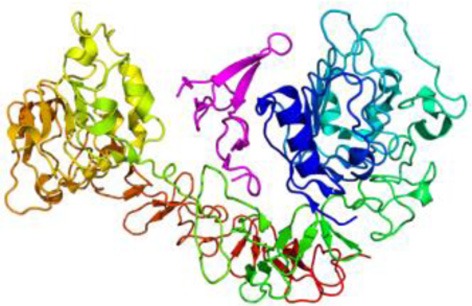
ErbB2, HER2 or herstatin;	185 KDa	ErbB2 is the only ErbB family member that does not bind a known ligand. It works as a signal transducer following ligand dependent recruitment into heterodimer	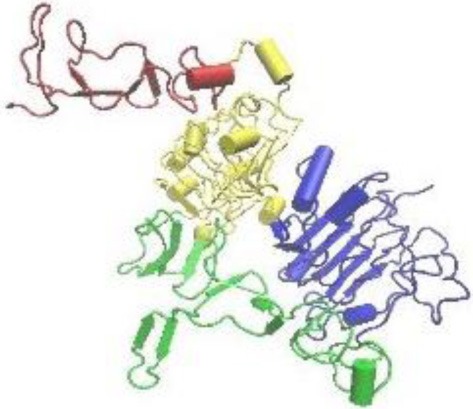
		rs with ErbB1, ErbB3, or ErbB4	
ERbB3 or HER3	140 kDa	ErbB3 consists of four domains with structural homology to domains found in the type I insulin-like growth factor receptor. ErbB3 has a heregulin (HRG) or neuregulin binding domain but lacks intrinsic protein tyrosine kinase activity. Therefore, it can bind to the ligand but not convey the signal into the cell through protein phosphorylation. However, ErbB3 forms heterodimers with other EGF receptor family members which do have kinase activity	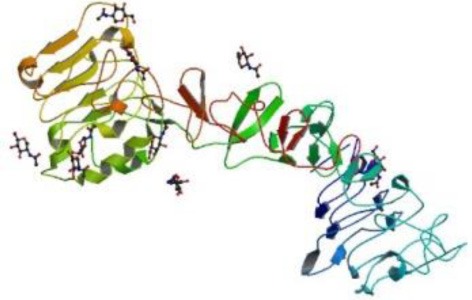
ErbB4, HER4 or V-Erb-A Erythroblastic Leukemia Viral Oncogene Homolog 4	200 kDa	ERBB4 is a unique member of the ERBB family that can undergo regulated intramembrane proteolysis. ERBB4 is a single-pass type-1 transmembrane protein with multiple furin-like cysteine rich domains, a TK domain, a PI3K binding site and a PDZ domain binding motif. This receptor is activated by neuregulins-2 and -3, heparin-binding EGF-like growth factor and betacellulin	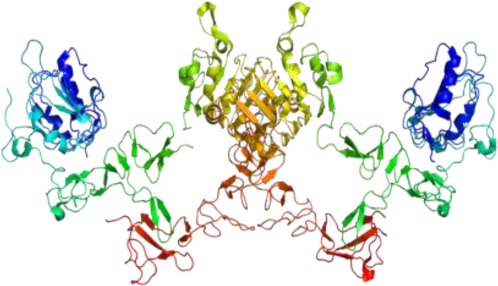

Molecular subdomains are represented by colors: L1 (blue), L2 (yellow), CR1 (green), and CR2 (red).

The EGFR is synthetized as a precursor molecule that is integrated by a 1210-residue polypeptide. After cleavage in the N-terminal domain, a final fragment of 1186-amino acid protein is anchored on the cellular membrane surface (Jorissen et al., 2003). EGFR has a small transmembrane hydrophobic region and a tyrosine kinase intracellular domain (Yarden and Sliwkowski, 2001; Herbst and Bunn, 2003; Scaltriti and Baselga, 2006). The molecular structure of the EGFR is also characterized for four domains abundant in cysteine-rich (CR1 and CR2) and leucine-rich residues (L1 and L2 domains), which are the ligand binding regions (Jorissen et al., 2003; Flynn et al., 2009). EGFR ligands are the epidermal growth factor (EGF), heparin-binding EGF-like growth factor (HB-EGF), β-cellulin, transforming growth factor alpha (TGF-α), amphiregulin (AREG), epiregulin (EREG), epigen (EPGN) and neuroregulin (NRG). All these ligands can activate the EGFR by autocrine or paracrine signaling (Schneider et al., 2008). The most important ligand of the EGFR is the EGF, a 5.5 kD peptide discovered in the 50s by Stanley Cohen when he was trying to purify the nerve growth factor (NGF) (Raivich and Kreutzberg, 1994; Nathoo et al., 2004). The EGF binds to high and low affinity sites in EGFR-expressing cells. Truncation of the CR1 domain, a loop that mediates dimerization, abolishes the high-affinity binding cell population (Jorissen et al., 2003). Yet, the precise mechanism that regulates these affinities is still unknown.

## Signaling cascades of the ErbB receptor family

**Figure 4:**
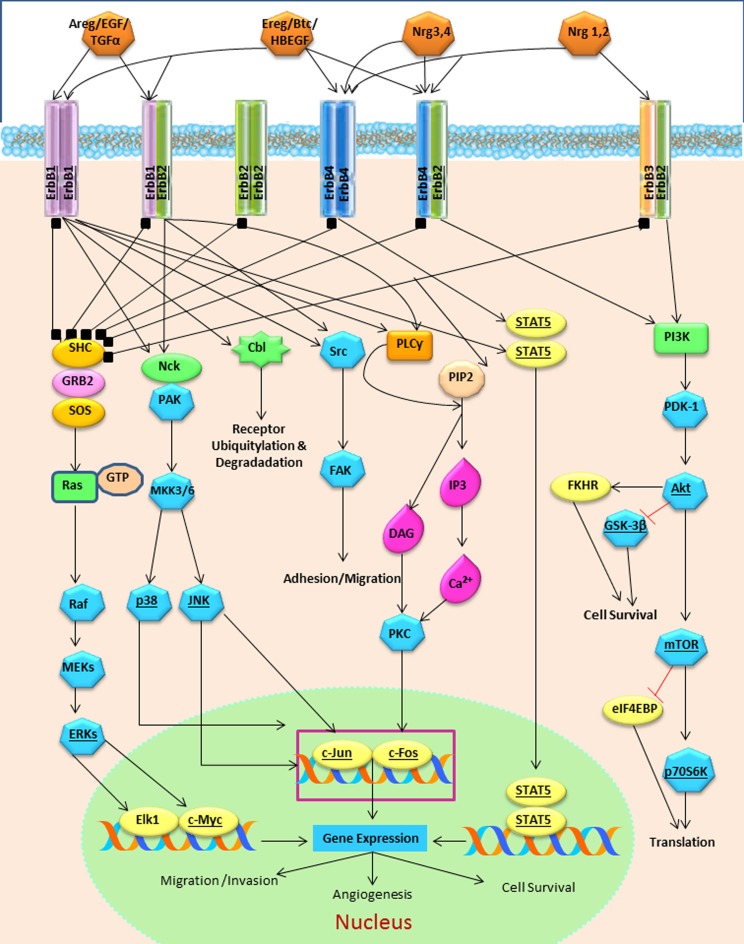
**The ErbB family receptors and their main cell signaling pathways: the Ras/MAPK, the PI3K/AKT and the PLCy pathways**.

**Figure 5:**
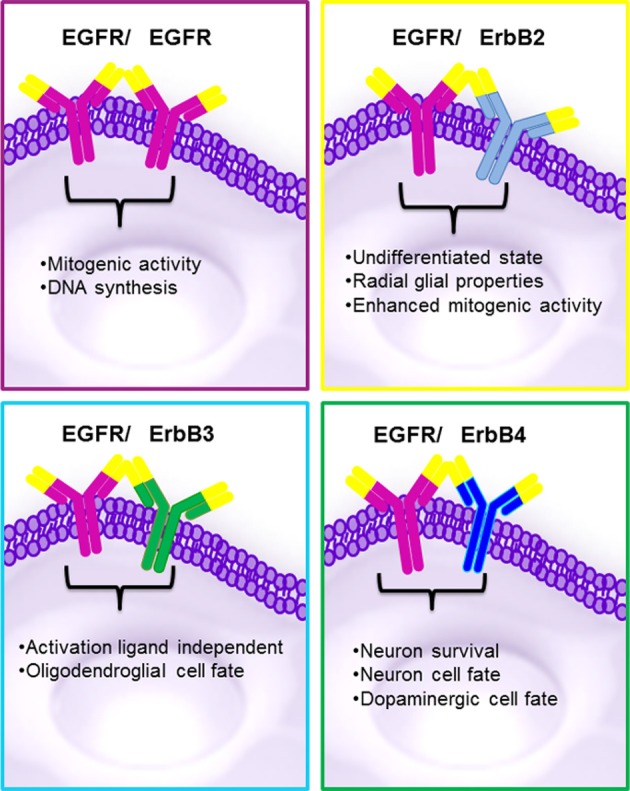
**Biological effects reported in ErbB family proteins.** The homo or heterodimerization of the ErbB proteins may generate similar effects. Homodimerization between EGFR/EGFR can originates proliferation MAPK activation and the heterodimeriizations of EGFR/ErbB2 undifferentiated state and enhanced mitogenic activity; EGFR/ErbB3 oligodendroglial cell fate via AKT and EGFR/ErbB4 neuronal and survival cell fate.

The strength and intensity of ErbB signaling depends principally on two biological mechanisms: (1) the homodimerization or heterodimerization among ErbB family receptors, and (2) the subsequent activation of downstream proteins (Jorissen et al., 2003; Clark et al., 2012). The ErbB receptor family may signal through PI3K/Akt (phosphoinositide 3-kinase/ v-akt murine thymoma), MAPK (the mitogen-activated protein kinases), STAT, PLC (phospholipase C) and many other pathways (Figure 4), thereby, regulating cell proliferation, apoptosis, differentiation or migration (Nathoo et al., 2004; Quesnelle et al., 2007). Following activation of EGFR, the SH2 domain of Grb2 can bind to the EGFR either directly (via Y1068 and Y1086) or indirectly via tyrosine phosphorylated Shc (Sasaoka et al., 1994). The association of Shc to EGFR through its PTB domain recruits Grb2 and constitutes one of the main steps in EGF-dependent induction of the Ras/MAPK pathway (Gong and Zhao, 2003). Heterodimerization between different ErbB family members generates a number of cellular effects (Figure 5). For instance, heterodimerization between ErbB1 and ErbB2 triggers mitosis (Yarden, 2001) and an undifferentiated cell stage (Ghashghaei et al., 2007). Heterodimerization between EGFR/ErbB4 is involved in dopamine neurons development (Iwakura et al., 2005), whereas ErbB2/ErbB3 is implicated in oligodendrocyte differentiation (Makinodan et al., 2012) by activation of PI3K/AKT pathway (Clark et al., 2012). Interestingly, ErbB2/ErbB3 heterodimerization can also promote glioma development (Clark et al., 2012). ErbB2/ErbB4 promotes cell differentiation in the embryonic hippocampus (Gerecke et al., 2004). ErbB1/ErbB4 activation mediated by betacellulin expands neural stem cells and neuroblasts in the postnatal brain (Gomez-Gaviro et al., 2012). In summary, the ErbB receptor family is involved in a myriad of biological effects, one of them is the generation and differentiation of oligodendrocytes from neural stem cells.

## Oligodendrocyte specification mediated by ErbB family in the embryonic brain

In embryonic neural precursors, the EGFR is mainly associated with initiation of asymmetric divisions (Sun et al., 2005). The ErbB family regulates the maturation process of oligodendrocytes and myelin production during neural development (Aguirre et al., 2007). In rodents, gliogenesis occurs in the second week of postnatal development (Ivkovic et al., 2008), which is associated with the EGFR expression (Aguirre et al., 2007). ErbB1 and ErbB2 mRNAs are expressed in S100β+ and Olig2+ glial precursors, while ErbB3 mRNA expression coincides with the expression of 2′,3′-cyclic-nucleotide 3′-fosfodiesterase (a marker associated with mature oligodendrocytes) (Abe et al., 2009). Furthermore, ErbB2 appears to participate in the oligodendrocyte differentiation process, while the expression of ErbB3 and ErbB4 is necessary for maturation of embryonic oligodendrocyte precursors (Sussman et al., 2005). Thus, a possible interaction between ErbB1 and ErbB3 signaling pathways may be involved in oligodendroglial specification.

AKT signaling is involved in myelination during early embryonic stages (Flores et al., 2008). SHP2 protein is involved in the AKT activation promoted by EGFR in oligodendrocyte precursors (Liu et al., 2011). Another targeting protein for the AKT signaling is Gab1; when Gab1 levels decrease in precursor cells of the spinal cord, the Olig-2 transcription factor expression decreases and the Pax7+ expression is inhibited (Hayakawa-Yano et al., 2007). This suggests that ErbB1 drives the Olig-2 expression via Gab1/AKT.

Neuroregulins (NRGs) are efficient ligands for EGFR, ErbB3, and ErBb4 proteins (Talmage, 2008; Clark et al., 2012). NRGs support the survival of oligodendrocytes and aged oligodendrocyte precursor cells (Fernandez et al., 2000). NRGs induce axon-associated survival in developing oligodendrocytes through the PI3-kinase/Akt pathway (Flores et al., 2000). Inhibition of the signaling mediated by NRG/ErbB4, an important regulator of oligodendrocyte development, induces changes in the morphology, number and role of the oligodendrocytes *in vivo* (Roy et al., 2007). ErbB family members also promote myelination in the peripheral nervous system by increasing the expression of myelin protein zero (P0 or MPZ) in Schwann cells (Chen et al., 2006). Neuroregulin 1 (NRG-1) induces heterodimerization between ErbB2 and ErbB3 receptors and promotes myelination in the peripheral nervous system (Newbern and Birchmeier, 2010). Taken together this indicates that NRGs play an important role in oligodendrogenesis and myelination during CNS and PNS development.

## ErbB effects in the oligodendrocyte specification in adult neural stem cells

Increasing evidence indicates that EGFR ligands determine the cell fate of adult neural stem cell of the VZ-SVZ (Gonzalez-Perez and Alvarez-Buylla, 2011). At the postnatal day 3, EGFR overexpression in the VZ-SVZ generates intense hyperplasia. These cells express oligodendrocyte-type lineage markers characterized by the expression of NG2, Olig2, and PDGFRα (Ivkovic et al., 2008). In the adult VZ-SVZ, intracerebral administration of EGF induces strong cell proliferation and migration and drives differentiation into oligodendrocyte lineage (Gonzalez-Perez et al., 2009). In cell culture, VZ-SVZ astrocytes (type B cells) strongly proliferate and generate a number of O4+PDGFRα+ oligodendrocyte precursors after EGF exposure (Gonzalez-Perez and Quinones-Hinojosa, 2010). The astrocytic cells respond to the EGFR stimulation through several ligands and MAPK signaling pathway (Tournier et al., 1994). Interestingly, after three weeks in cell culture astrocytes do not respond to EGF stimulation, but when pre-treated with IL-6, astrocytes actively respond to EGF effects (Levison et al., 2000; Gonzalez-Perez et al., 2012). These findings indicate a synergy between IL-6 and EGF. A similar effect is also observed in spinal cord progenitor cells, where IL-6 produces a trans-activation of EGFR (Kang and Kang, 2008). IL-6 activates Jak/STAT signaling pathway (Scaltriti and Baselga, 2006) and stimulates oligodendroglial differentiation via the Jak/STAT pathway (Islam et al., 2009). STAT-3 is essential for the synergism between the chondroitin sulfate proteoglycan (CSPG) and EGF. In cell culture, CSPG and EGF activate PI3K and STAT-3 pathways, as well as promote the formation of neurospheres, cell survival and the phosphorylation of EGFR (Tham et al., 2010). The PI3K/AKT pathway is mainly activated when EGFR dimerizes with the ErbB3 monomer (Scaltriti and Baselga, 2006). EGFR-dependent STAT activation may be mediated by Src without JAK. Src is involved in the activation of PI3K/AKT via a p85 subunit binding (Jorissen et al., 2003) (Figure 4). Taken together, this suggests that Jak/STAT is important for cell-cycle progression and oligodendrogenesis.

ADPbetaS and UTP nucleotides activate EGFR and induce a rapid calcium influx in SVZ progenitor cells (Grimm et al., 2009). An increase in intracellular calcium levels promotes oligodendrocyte differentiation (Boscia et al., 2012; Paez et al., 2012). Oligodendrocyte differentiation and myelination is promoted by the NMDAR stimulation and by subsequent influx of calcium (Cavaliere et al., 2012). NRG/ErbB induces oligodendroglial differentiation (Roy et al., 2007) probably mediated by NMDAR activation (Brinkmann et al., 2008). The interaction between the ErbB and NMDAR during oligodendrocyte differentiation is mediated by intracellular levels of calcium (Brinkmann et al., 2008; Cavaliere et al., 2012). This indicates that the synergism between EGF and calcium ion channels controls oligodendrocyte specification.

Polidendrocytes, also known synantocytes or NG2 glia, are characterized by the expression of the NG2 chondroitin sulfate proteoglycan (Nishiyama, 2001). Many of the NG2-expressing cells are located in the corpus callosum and express the EGFR. In wild-type mice, re-myelination is mediated by NG2+Olig2+Mash1+ precursor cells (Aguirre et al., 2007). In contrast, mutant mice that constitutively express the human EGFR (hEGFR) in NG2 cells show an increase in the expression of Nkx2.2, Sox-9, and Sox-10 in a demyelinated area. Thus, an increase in the activity of EGFR drives oligodendrogenesis at the expense of astrogliogenesis (Aguirre et al., 2007). Consequently, the transcription factors Nkx2.2, Sox-9, and Sox-10 may regulate the oligodendrogenesis effects of EGFR in multipotent cells. Sox-2 is a transcription factor that has homology with the SRY protein and determines the sex of individuals (Hu et al., 2010). Sox-2 is associated with oligodendrocyte-cell-fate commitment in the SVZ and SGZ precursor cells that express EGFR (Komitova and Eriksson, 2004; Baer et al., 2007; Balu and Lucki, 2009). Interestingly, changes in EGFR gene expression and EGFR protein occur at the time of puberty in the brain (Ma et al., 1994) and sex-dependent differences have been observed in myelination process (Kipp et al., 2012). Nevertheless, the biological relationship between ErbB family receptors and sexual hormones in oligodendrocyte specification remains to be elucidated.

Olig1 and Olig2 transcription factors participate in oligodendrogenesis in embryonic spinal cord and ventral telencephalon (Jakovcevski and Zecevic, 2005a,b). In the adult brain, a small population of type-B cells in the SVZ expresses Olig2 that may generate a discrete subpopulation of Olig2-expressing type-C cells. These Olig2+Dlx2+ type-C cells may be the oligodendroglial precursors of adult VZ-SVZ (Menn et al., 2006; Gonzalez-Perez and Quinones-Hinojosa, 2010). Interaction between Dlx2 and EGFR regulates proliferation and neurogenesis of type C cells in the VZ-SVZ (Suh et al., 2009).

MAPK pathway promotes neuronal differentiation and survival through phosphorylated CREB (CREB-p) that, in turn, induces Pax6 expression (Levison et al., 2000; Gampe et al., 2011; Herold et al., 2011; Yoo et al., 2012). Pax-6 reduces the EGFR expression in neural stem cells (Jia et al., 2011) and represses the expression of Olig-2 cells and glial lineage (Jang and Goldman, 2011). Thus, Pax-6 induces neuronal differentiation in embryonic and adult neural precursor cells (Kohwi et al., 2005; Osumi et al., 2008) and determines the neuronal cell fate while decreasing the expression of NG2 (Klempin et al., 2011). Hence, interaction between Pax-6 and Olig-2 seems to regulate the final phenotype of precursor cells by regulating EGFR activity. This intricate relationship among transcription factors and downstream signaling pathways activated by ErbB proteins regulates the oligodendrogenesis process. Therefore, molecular disruptions in any of these biological mechanisms may considerably alter the myelination process. ErbB-driven olidodendrogenesis has been implicated in psychiatric disorders and tumorigenesis.

## ErbB receptors in psychiatric disorders

Disruption in oligodendrogenesis and myelin formation has been associated with the pathophysiology of schizophrenia. Animals deprived of ErbB signaling show psychiatric-like behaviors (Roy et al., 2007). The brain of schizophrenic patients shows a significant alteration in myelination and oligodendrocyte generation (Chang et al., 2007), which is associated with a decrease in EGF levels (Futamura et al., 2002; Iwakura and Nawa, 2013). Mature oligodendrocytes express the dopamine receptors (D2 and D3) that are involved in myelin-sheath formation and oligodendrocyte turnover. A deficiency in expression of D2 and D3 receptors reduces the number of myelinating oligodendrocytes (Lee and Fields, 2009). In contrast, dopamine exposure promotes EGFR trans-activation, which increases the proliferation of neural precursor cells in the adult brain (Winner et al., 2009; O'keeffe and Barker, 2011). Therefore, ErbB receptor family is an important regulator of dopamine-induced oligodendrogenesis, which may explain some of the myelination disturbances observed in schizophrenia.

In major depression disease, a low density and reduced expression of oligodendrocyte-specific gene transcripts have been found in postmortem human brains (Edgar and Sibille, 2012). Patients with major depression often present with decreased levels of EGF (Tian et al., 2012). Oligodendrocyte precursor cells co-express serotonin receptors and EGF receptors (Schaumburg et al., 2008), while EGF has been shown to transcriptionally regulate transporters of serotonin 1 and 2 via ErbB1 (Gill et al., 2011) Taken together, this evidence suggests that ErbB receptors may be involved in the pathophysiology of mood and psychiatric disorders by controlling the oligodendrocyte cell population and serotonin brain levels.

## EGFR role in hyperplasia and tumorigenesis

In the adult brain, long-lasting EGFR stimulation induces polyp-like growths (Kuhn and Miller, 1996), leads to diffuse white-matter hyperplasia (Ivkovic et al., 2008) and promotes a strong invasive pattern of VZ-SVZ precursors (Gonzalez-Perez et al., 2009; Gonzalez-Perez and Quinones-Hinojosa, 2010). Remarkably, in all these experiments, the EGF-transformed cells appear to be related to oligodendroglia cell lineage, such as: NG2, Olig-2, O4, and PDGFRα. In brain tumors, there is evidence suggesting that EGFR signaling plays a role in tumor initiation and progression. Approximately 50% of high-grade astrocytomas show EGFR amplification that leads to glioblastoma malignization (Maher et al., 2001; Wechsler-Reya and Scott, 2001). Low-grade and high-grade oligodendroglial tumors also show an increased expression of EGFR mRNA and protein, which in the vast majority of cases is not produced by gene amplification (Reifenberger et al., 1996). Furthermore, EGFR overexpression is associated with mutations in the intracellular and extracellular domains, alterations in signaling cascade regulation and cross-signaling with other tyrosine kinase receptors (Schneider et al., 2008). This indicates that the EGFR may be activated by ligand-dependent and ligand-independent mechanisms (Scaltriti and Baselga, 2006). The amplification and overexpression of EGFR is found in approximately 50% of glioblastomas and promotes invasion, proliferation and apoptosis arrest (Clark et al., 2012). These effects are produced by AKT and ERK 1/2 (Clark et al., 2012). In approximately 14% of EGFR-expressing glioblastomas there is a mutation in the EGFRvIII gene, which is characterized by a lack of an extracellular domain and constitutive phosphorylation (Brandes et al., 2008). The EGFR contributes to the tumorigenic cellular diversity of glioblastoma by participating in the expansion of diverse cell types that initiate tumors (Mazzoleni et al., 2010). Whether *stem-cell-like* glioblastoma cells are initially exposed to EGF and bFGF, the EGFR-like activity continues despite the absence of a ligand. This suggests that other erbB family members, i.e., erbB2 and erBb3, may also be involved in glioblastoma cell expansion (Clark et al., 2012). Interestingly, EGFR overstimulation by itself is not enough to drive brain tumor formation. This indicates the genetic background plays an important role in tumorigenesis and the EGFR may only induce certain aspects of a malignant phenotype (e.g., proliferation and tissue invasion).

## Conclusion

**Figure 6:**
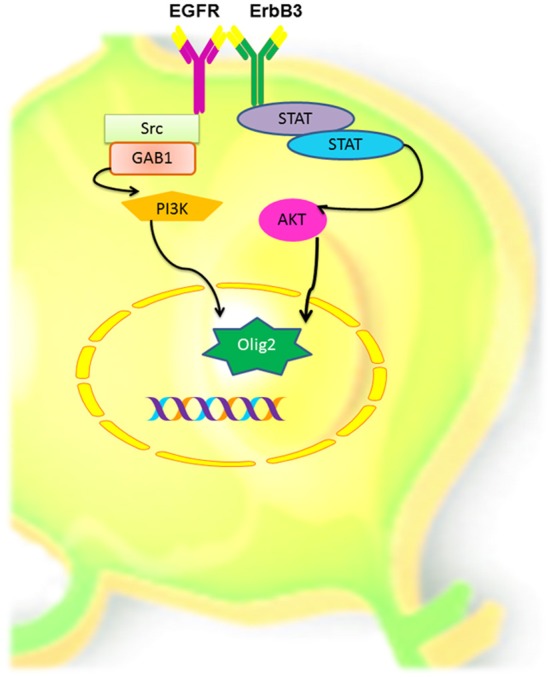
**Hypothetical model of oligodendrocyte cell signaling, via EGFR and ErbB3 in adult neural stem and progenitors cells.** Homodimerization between these two ErbB members could activate the PI3K or the STAT pathways that in turn can activate AKT and induces the expression of Olig-2, which in turn may determine oligodendroglial lineage.

EGFR has different biological effects that can be attributed to: (1) the type of ErbB ligand (Jin et al., 2002; Cooper and Isacson, 2004; Gonzalez-Perez et al., 2009; Gonzalez-Perez and Quinones-Hinojosa, 2010; Gomez-Gaviro et al., 2012); (2) the homo or heterodimerization ErbB proteins (Iwakura and Nawa, 2013) and (3) the downstream signaling pathway that every ligand/dimmer may stimulate. ErbB proteins are important mediators of quiescence, proliferation, differentiation, phenotype specification, survival and migration of neural stem cells. In a number of independent reports, ErbB1 and Erbb3 are frequently involved in oligodendrocyte specification. Therefore, we hypothesize that the interaction between these receptors is one of the main promoters in the oligodendrocyte lineage (Figure 6). Yet, the role of the ErbB family members in adult neural stem cells is not completely understood. Elucidating the precise role of ErbB family members in oligodendrogenesis is a crucial step for designing stem-cell-based therapies for demyelinating diseases and other neurological disorders.

### Conflict of interest statement

The authors declare that the research was conducted in the absence of any commercial or financial relationships that could be construed as a potential conflict of interest.
